# Subglacial discharges create fluctuating foraging *hotspots* for sea birds in tidewater glacier bays

**DOI:** 10.1038/srep43999

**Published:** 2017-03-07

**Authors:** Jacek Andrzej Urbanski, Lech Stempniewicz, Jan Marcin Węsławski, Katarzyna Dragańska-Deja, Agnieszka Wochna, Michał Goc, Lech Iliszko

**Affiliations:** 1 GIS Centre, University of Gdansk, 80-952 Gdansk, Poland; 2 Department of Vertebrate Ecology and Zoology, University of Gdansk, 80-308 Gdansk, Poland; 3 Institute of Oceanology, Polish Academy of Sciences, 81-712 Sopot, Poland

## Abstract

Although the processes occurring at the front of an ice face in tidewater glacier bays still await thorough investigation, their importance to the rapidly changing polar environment is spurring a considerable research effort. Glacier melting, sediment delivery and the formation of seabird foraging hotspots are governed by subglacial discharges of meltwater. We have combined the results of tracking black-legged kittiwakes *Rissa tridactyla* equipped with GPS loggers, analyses of satellite images and *in situ* measurements of water temperature, salinity and turbidity in order to examine the magnitude and variability of such hotspots in the context of glacier bay hydrology. Small though these hotspots are in size, foraging in them appears to be highly intensive. They come into existence only if the subglacial discharge reaches the surface, if the entrainment velocity at a conduit is high and if there is sufficient macroplankton in the entrainment layer. The position and type of subglacial discharges may fluctuate in time and space, thereby influencing glacier bay hydrology and the occurrence of foraging hotspots.

Tidewater glaciers and the waters of bays in front of ice faces are unique features of arctic fjords. Recent years have witnessed greater research efforts in these areas, as glacier retreat is one of the most spectacular, visible signs of global warming^1^. Glacier bays play a significant part in sedimentation, ice cover formation and iceberg calving, thus bringing a substantial influence to bear on the ecology of fjord coastal waters^2^,^3^. Because glacier termini are accessible only with great difficulty, very few field measurements have been carried out there, and then only at some distance from the ice wall. Certain processes have therefore been investigated only in the laboratory or using mathematical models. Several geophysical processes take place near or on the ice face of tidewater glaciers. Ice-front melting due to contact with seawater creates constant upwelling along the ice face. Some authors suggest that this process is negligible when compared with the terminus retreat rate^4^,^5^, but others have attempted to demonstrate that it may play an important role^6^,^7^. The fluvial discharge of meltwaters is another process generally regarded as crucial to the hydrology and ecology of glacier bays. A discharge outlet may be situated beneath the glacier (subglacial discharge), somewhere on the ice face itself (englacial discharge) or on the surface of the glacier (supraglacial discharge). There may be many outlets of different kinds on the glacier front, and the position and volume of discharged water may differ in time as a consequence of varying weather conditions and changes in ice face shape and movement^5^.

Subglacial freshwater discharge has been shown to be the primary process driving high rates of submarine melting in tidewater glaciers^8^,^9^,^10^, which has the potential to control terminus morphology and calving style^11^. The delivery to a fjord system of fluvial freshwaters, turbid as a result of the large concentrations of suspended matter they carry, creates muddy plumes close to the glacier front and involves a huge flux of sediments^2^,^5^,^12^. The biological importance of subglacial discharges is reflected in the shallower euphotic zone, changes to water parameters like salinity and temperature, and the entrainment of organisms and nutrients carried to the glacier front by the rising plume^13^,^14^.

The significance of submarine ice melting is most often described using upwelling or buoyant convective plume models^6^,^10^,^12^,^15^,^16^, whereas in research into sediment distribution, submarine discharges take the form of a buoyant jet described as a forced plume whose behaviour depends on the density difference between the plume and seawater and on the jet’s momentum. The instant subglacial meltwater leaves a conduit at the glacier front, intensive mixing of the plume with ambient marine waters takes place. The resulting buoyant plume rises until it reaches the surface or a layer of water with a density equal to that of the plume, after which it begins to flow horizontally. In stratified waters, therefore, a turbid subglacial plume may never reach the surface^5^,^10^. The plume grows as a result of turbulent mixing with warmer ocean waters and drives locally intensified melting at the ice-ocean interface^15^, which may lead to the formation of ice caves, reported to be tens of meters wide and deep^5^. Moreover, if the jet’s momentum is the dominant force, superelevation of the jet may occur on the surface, a phenomenon known as water “boiling”^5^ (Supplementary Video S2).

A number of specific ecological effects of meltwater discharge can be observed in the vicinity of the glacier terminus. The water area around the subglacial discharge that is ice-free due to currents and muddy due to suspended sediments is called the “brown zone”^17^ and is considered an attractive foraging site for seabirds^13^, seals^18^ and white whales, which feed mainly on polar cod^19^. Large amounts of marine zooplankton, stunned or killed by osmotic shock, may be found in such zones^20^,^21^,^22^,^23^. Meltwater discharges also affect the euphotic depth in glacier bays, which has a direct impact on phytoplankton growth^13^. The type of microplankton near the glacier front changes from autotrophic to heterotrophic, and bacteria increase in number, possibly because of the presence of fine mineral particles acting as nuclei of microbial aggregates^13^. Large numbers of dead zooplankton are known to sink to the seabed near glacier cliffs^21^, and several carrion-feeding benthic organisms take advantage of this resource^23^. However, large concentrations of live macroplankton (euphausiids) have also been recorded on the seabed near glaciers, apparently feeding there^23^. The presence of highly saline, winter-cooled water is typical of the bottom layer of glacier bays in the Arctic, which produces the conditions preferred by the cold-water stenotherms often found there^13^. Altogether, the ecology of glacier bays is governed by the advection of biomass-rich waters from the shelf and their interaction with the topography and hydraulic forces near the glacier.

Although the vast marine environment appears to be homogeneous, the macroinvertebrates and fish that constitute the bulk of food taken by seabirds and marine mammals are patchily distributed. They are concentrated in certain specific areas like shelf slopes, ice edges, oceanographic fronts and upwelling sites, where physical gradients enhance the abundance of prey and its availability to seabirds in several ways. These areas are conspicuous by their greater abundances of marine birds and mammals^24^. Glacier bays with intensive subglacial discharges are just such areas and are well known as attractive foraging grounds for seabirds like black guillemots *Cepphus grylle*, ivory gulls *Pagophila eburnea* and especially kittiwakes *Rissa tridactyla*. Large aggregations of these have been observed in various parts of Svalbard in the previously mentioned “brown zones” of glacier meltwater outlets. Marine mammals, such as ringed seals *Pusa hispida*, bearded seals *Erignathus barbatus*, belugas *Delphinapterus leucas* and polar bears *Ursus maritimus* also occur there in much higher numbers than in other fjord habitats^13^. The large supply of easily available food is thought to be a reason for these concentrations of marine vertebrates in the glacier bays, although the mechanism by which such foraging hotspots form is still a matter of intensive discussion^13^. For foraging, however, seabirds use only a relatively small part of the “brown zone” close to the glacier river discharge and along the border between the fresh and saline waters. As a result of wind and wave activity, the “brown zone” is often broken up into separate long strips moving over the water surface far away from the glacier bay. Northern fulmars *Fulmarus glacialis* are often observed foraging along them^25^.

This study was motivated by a series of observations of kittiwake aggregations foraging on the muddy, subglacial waters discharged by glacier termini. In Kongsfjord in 2015 we noted huge numbers of kittiwakes, from several hundred to a few thousand, foraging in front of the differently sized ice caves at each glacier terminus in the fjord (Fig. 1). Drone video of kittiwakes foraging in front of Storbreen in Hornsund can be found as Supplementary Video S2. Present knowledge and understanding of the processes at tidewater glacier fronts is still unsatisfactory, and a number of questions remain unanswered: Do measurements confirm the theory of physical processes of subglacial water discharge? What is the mechanism of plankton flux to foraging hotspots? How large are the bird aggregations and how important are these particular foraging areas for seabirds during the chick-rearing season? What influences the spatial and temporal distribution of such hotspots? Why do not all water discharges in glacier bays influence foraging aggregations? We set up three hypotheses in an attempt to find answers to these questions: 1) the muddy waters close to the glacier front occasionally create important foraging areas for birds during the chick-rearing season; 2) discharges of subglacial water sometimes form relatively small hotspots with large amounts of dead or stunned macroplankton on the surface of waters in front of the ice caves; 3) these hotspots are associated with turbid fluvial discharge waters and come into being during the entrainment of the seawaters surrounding the subglacial discharge where macroplankton occurs. We tested these hypotheses experimentally by combining the results of tracking black-legged kittiwakes *Rissa tridactyla* (henceforth kittiwakes) equipped with GPS loggers with the results of coupling satellite images and *in situ* measurements of water temperature, salinity, turbidity and macroplankton in glacier bays.

## Study Area

**Figure 1:**
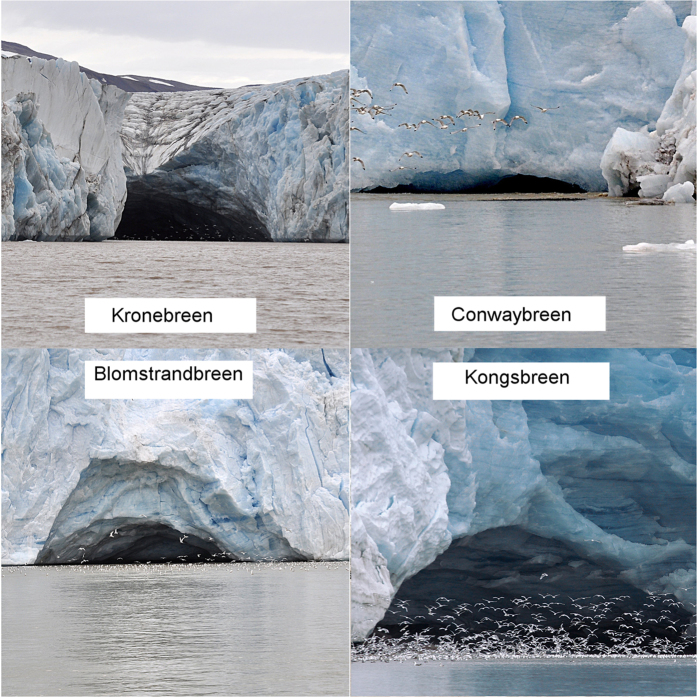
Observations of seabird aggregations. Seabirds were observed near ice caves with subglacial discharges at glacier termini in Kongsfjord in August 2015. The estimated number of kittiwakes in front of Kongsbreen was 1500. The positions of the glaciers are shown on Fig. 2.

**Figure 2:**
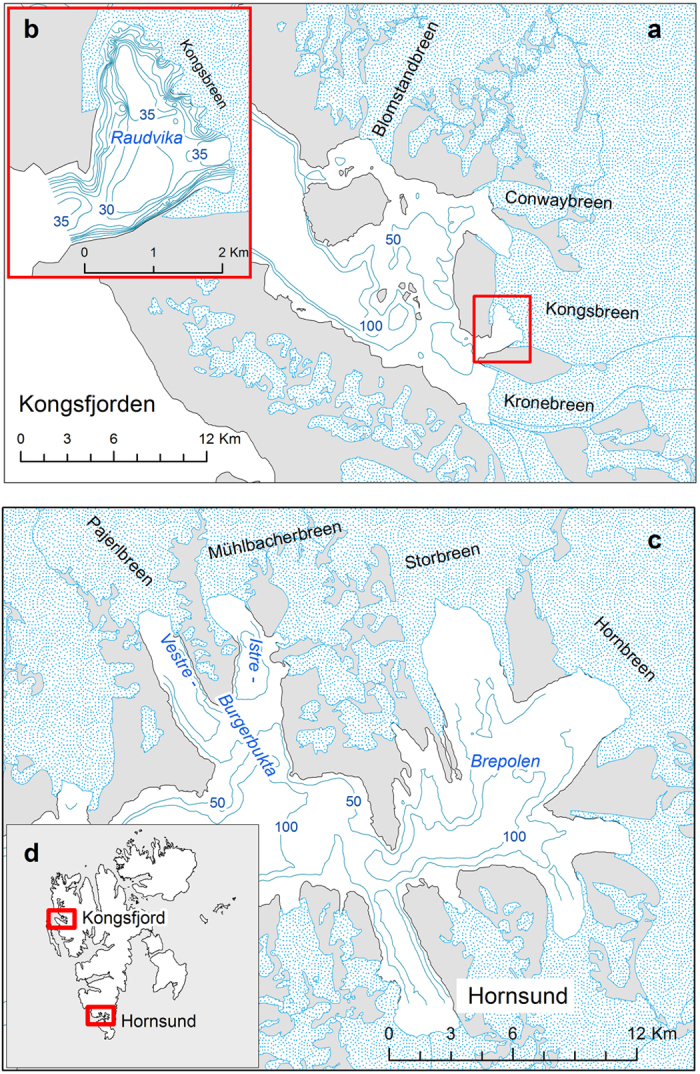
Project location map. Kongsfjord and Hornsund (**a**,**c**) are situated on the west coast of Spitsbergen, the largest island in Svalbard (**d**). In Kongsfjord (**a**) the area of interest was Raudvika bay, outlined by the red rectangle. The contours in Raudvika (**b**) are drawn at 5 m intervals. In Hornsund (**c**) measurements were carried out in Burgerbukta and its branches to Vestre Burgerbukta and Austre Burgerbukta. The depth contours on the maps (**a**,**c**) are drawn for 50 and 100 m depths only. The maps were drawn in ArcGIS 10.3.1 for Desktop, Esri Inc., http://www.esri.com.

We chose two fjords – Kongsfjord and Hornsund – on the west coast of Spitsbergen, each with several glacier bays having trunk tidewater glacier termini (Fig. 2). The Kongsfjord is probably the best described fjord in Svalbard, and subglacial discharges of meltwater have been recorded there several times^5^,^26^. There are large breeding colonies of kittiwakes in both fjords^13^. Kittiwakes were also recorded at glacier ice faces in Hornsund during the seabird and marine mammal surveys conducted by our team in Burgerbukta in 2014 and 2015^25^. Two bays: Burgerbukta and Raudvika were examined in closer detail. Burgerbukta splits into two narrow bays with tidewater glaciers at their ends; these bays are up to 100 m deep, but the depth near the glacier terminus is unknown because of its fast rate of retreat. Raudvika is a small glacier bay, formed in the last 20 years, and ends with the ice face of the Kongsbreen glacier. This bay was chosen because the water was ice-free, the surface turbidity across the bay was highly variable (which is important when calibrating satellite images) and aggregations of kittiwakes were observed in front of the glacier terminus (Fig. 1). The ice face is partly grounded in shallow water or on the coast, but most of the bay is between 20 and 40 m deep, with similar depths near the ice face. Both Burgerbukta and Raudvika have a sill at their mouths: this gives rise to the formation of a three-layered structure of the water masses, with winter-cooled water at the bottom^27^.

## Results

### Distribution of subglacial water discharges

**Figure 3:**
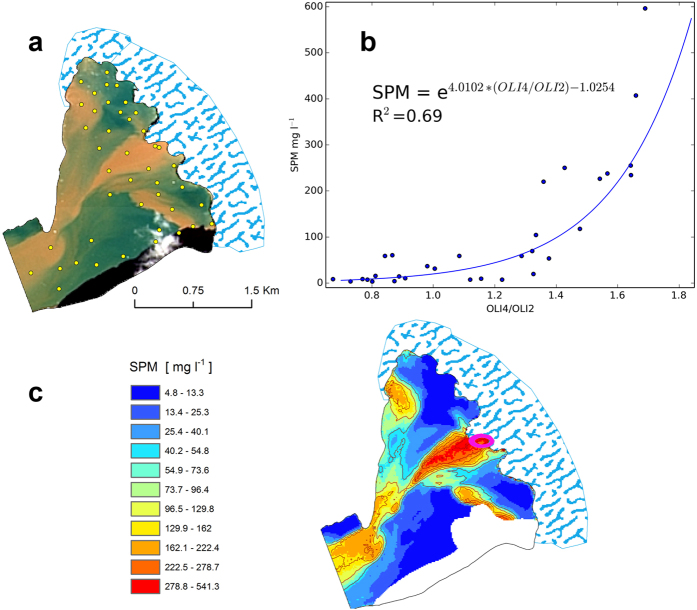
Mapping the surface distribution of suspended particulate matter in Raudvika. The composite of Landsat 8 satellite image bands shows different colours of the water as a result of different SPM levels at the surface. The sampling stations are shown by yellow circles (**a**). The correlation model was used to convert reflectance bands to SPM (**b**). The SPM map can be used to identify remote meltwater discharges reaching the surface. The magenta circle shows the aggregation of birds (1500 birds) (**c**). The maps were drawn in ArcGIS 10.3.1 for Desktop, Esri Inc., http://www.esri.com. The Landsat image was provided by: U.S. Geological Survey, Earth Resources Observation and Science (EROS) Center, 2016, Landsat products and services: EROS, Glovis Web page, accessed 20 June 2016 at http://glovis.usgs.gov/.

**Figure 4:**
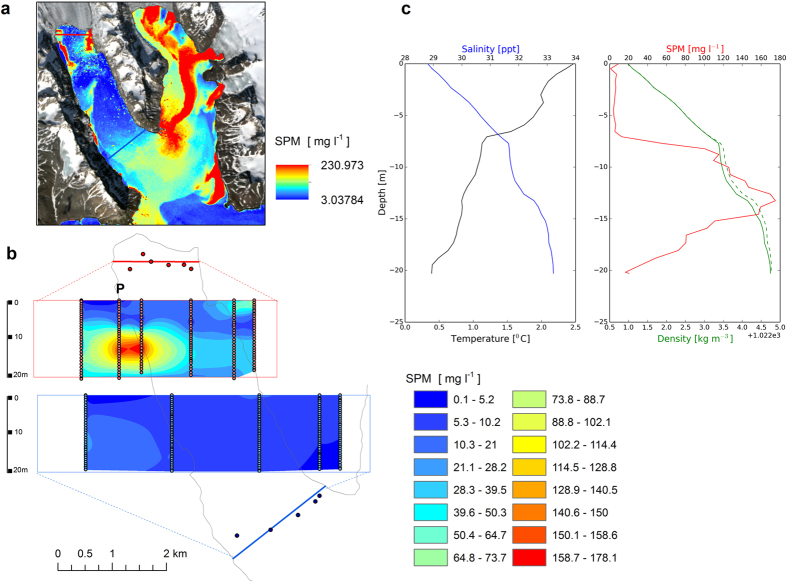
Subglacial discharges in Burgerbukta. The SPM distribution in Burgerbukta in Hornsund on 31 July 2015 (**a**) mapped from the Landsat 8 satellite image. Two SPM cross-sections were made using S, T, SPM profiles carried out at the same time (**b**). The T, S, SPM profiles and water density measured at point P on the cross-section closer to the glacier terminus are also presented; the density (dashed line) includes the influence of SPM (**c**). The maps were drawn in ArcGIS 10.3.1 for Desktop, Esri Inc., http://www.esri.com.

We used high resolution Landsat 8 satellite images taken on 8 August 2015 to map the surface distribution of suspended particulate matter (SPM) in Raudvika. The main challenge in such attempts is to make *in situ* measurements at the same time as the image is taken and to measure the background readings of the whole range of values occurring at that time. As Raudvika is relatively small and the SPM values there were highly variable (2–600 mg l^−1^), we were able to create a best-fitting regression model describing the relation between the reflectance ratio in bands 2 and 4 with measured SPM in that wide range, with the time gap between taking an image and making measurements being <3 h. The formula obtained was used to convert the multispectral image to the SPM map (Fig. 3); its universality with regard to other areas will be discussed later. Surface SPM near the glacier ice face was spatially variable. The main subglacial discharge in the middle of the glacier front had an SPM concentration >300 mg l^−1^ and a maximum width of about 300 m. A foraging hotspot with about 1500 kittiwakes was observed near the ice front where this discharge occurred. The waters along the ice face on both sides of the discharge were much less turbid, with SPM up to 15 mg l^−1^. At both ends of the glacier face, two melt water streams enter the bay, forming plumes with higher SPM levels. The trunk glacier bays can be characterised as low-energy environments with limited mixing, where the main forcing factors are tidal currents, gradient currents of meltwater discharge and stress due to katabatic winds. As a result, diverse yet patchy structures rather than a uniform layer form at the water surface. With this method we were able to remotely identify the meltwater discharges reaching the surface and to compare the subglacial discharges in two branches of Burgerbukta in Hornsund by coupling satellite SPM mapping and T,S,SPM measurements at two cross-sections: one about 200 m and the other about 3 km from Pajerlbreen (Fig. 4). The SPM surface mapping was carried out using a Landsat 8 image taken on 31 July, with SPM being calculated from bands 2 and 4 using the procedure described above. Using the same image, an SPM map was also drawn for Brepollen, the eastern part of Hornsund. The control measurements made at the same time in Vestre Burgerbukta were encumbered with a root mean square error (RMS) of 11 mg l^−1^. The surface SPM distributions in both Vestre and Istre Burgerbukta glacier bays were very different. In Vestre Burgerbukta high SPMs were recorded only in two streams of glacier meltwater at the ends of the glacier wall, whereas the SPM level in the water in front of Pajerlbreen was much lower. In Istre Burgerbukta by contrast, in front of Mülbrachenbreen, SPM concentrations were very high, indicative of subglacial discharge. Comparison of Istre Burgerbukta with Vestre Burgerbukta shows that the SPM concentration in the whole bay much larger (Fig. 4). The SPM cross-section in front of the Pajerlbreen glacier showed a plume of water with high SPM (up to 180 mg l^−1^) at a depth of about 14 m. This is indicative of a subglacial discharge in the form of a buoyant plume, which flows horizontally after having reached a density level of the water column equal to the plume density. The density profile for this bay shows uniform stability in the water column down to 20 m. The structure of the plume with a sharper upper edge is shown on the SPM profile. This plume was not observed in the second cross-section in the mouth of Vestre Burgerbukta (Fig. 4).

### Macroplankton in glacier bays

**Figure 5:**
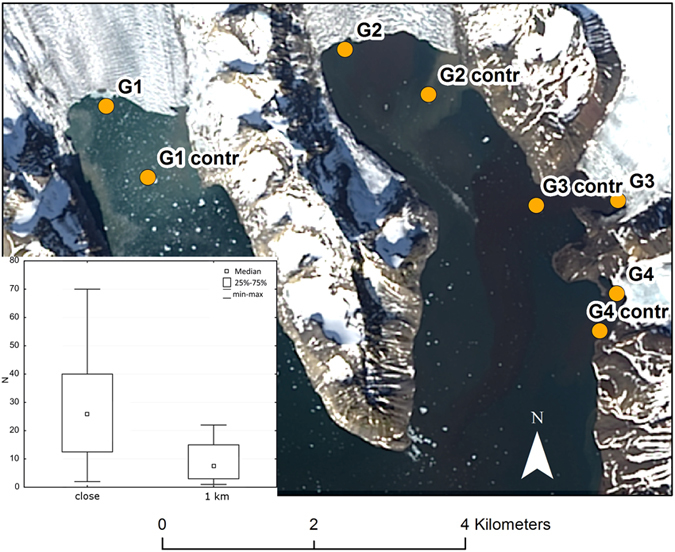
Macroplankton abundance in glacier bays. Macroplankton samples and near-surface macrozooplankton density (individuals per sample) – “close” – stations near the glacier cliff, 1 km – station away from the glacier. The Landsat image was provided by: U.S. Geological Survey, Earth Resources Observation and Science (EROS) Center, 2016, Landsat products and services: EROS, Glovis Web page, accessed 20 June 2016 at http://glovis.usgs.gov/.

There is a constant difference between the surface water macroplankton sampled in the two parts of Burgerbukta, i.e. close to the glacier cliffs and away from them: near the glacier they are more abundant and diverse than the controls (Fig. 5). Detailed results can be found in Supplementary Table S1. There was no taxon that was found only in the control samples but not near the glacier; on the other hand, there were several that were recorded near the glacier but not in the controls. This shows that the plankton recorded near the glacier is not a subsample of the fjord’s specific near-surface (neuston) macroplankton community but represents a selection of organisms living in the whole water column that were moved to the surface near the glacier. The influence of the glacier on surface plankton was especially strong in the case of large (10–30 mm long), actively swimming crustaceans (three species of herbivorous euphausiids *Thysanoessa* spp. and two species of carnivorous hyperiids *Themisto* spp.), which are represented much more abundantly in the near-glacier samples than in the controls. The difference between the control and glacier front samples is less marked in the case of poor swimmers like herbivorous copepods (0.2 to 2 mm long), and minimal in the case of passively mobile, carnivorous gelatinous plankton like *Ctenophora* and *Hydromedusae*. This shows that gelatinous plankton habitually occurs near the surface in the fjord and that the conditions close to the glacier have no effect on its occurrence pattern. Analysis of 25 food samples collected from Kittiwakes in the colony at the same time shows the dominance of Polar cod (84% samples), macroplanktonic euphausiids (44%) and hyperiids (24%).

### Seabird foraging hotspots at glacier fronts

In the deep tidewater glacier bays seabirds usually concentrated in the vicinity of the ice caves with their subglacial discharges of muddy waters. There are ice caves of different sizes at the fronts of most glacier termini in both Hornsund and Kongsfjord (Fig. 1). Some of them attracted from several hundred to many thousands of foraging kittiwakes. The highest number observed in this study was in July 2015, when around 10 000 individuals were foraging simultaneously along the c. 300 m frontline of Storbreen (personal obs.).

**Figure 6:**
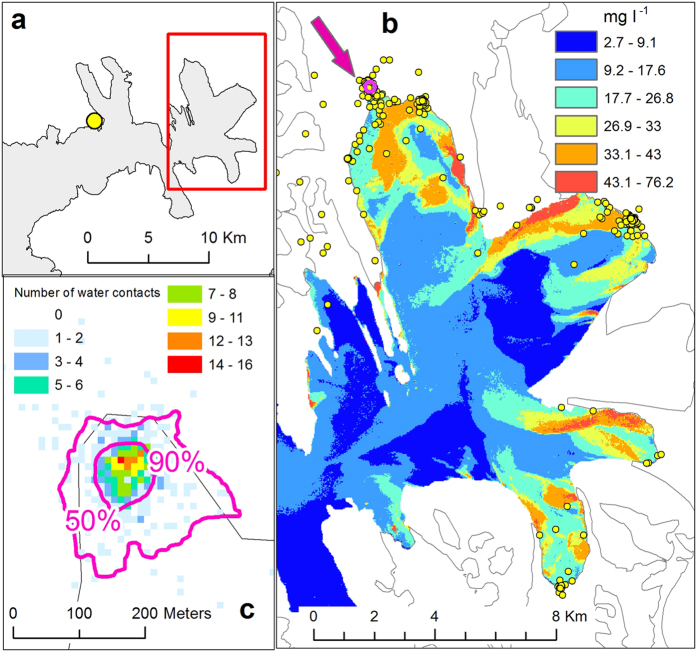
Kittiwake foraging hotspots. Foraging was recorded in 2015 in the Brepollen area, outlined as a red rectangle (**a**), for a sample of kittiwakes from a colony in Hornsund – yellow dot (**a**). Three foraging hotspots were recorded in Brepollen, where 36% of the kittiwakes’ foraging activity took place (**b**). The compactness of kittiwake distribution at the Storbreen ice face - magenta arrow at (**b**) - shows that foraging overlap more than 90% formed a hotspot about 40 m in radius (90% contour), while an overlap of over 50% covered an area with radius of about 100–200 m (50% contour) (**c**). (**c**) also shows the concentration of water contact points of Kittiwakes within the discharge plum. The maps were drawn in ArcGIS 10.3.1 for Desktop, Esri Inc., http://www.esri.com.

The distribution of the kittiwake sites in Brepollen is illustrated on Fig. 6. In 2015 several large aggregations of kittiwakes were observed. The majority of birds equipped with GPS loggers (9 out of 12 birds; 75%) visited this area during the study period and 36% of the 2 952 recorded body-water contacts took place in Brepollen. The mean time of the birds’ foraging events at the main hotspot in Brepollen was 3.3 ± SE 0.31 hour. Details of the GPS tracking study of kittiwakes in Brepollen are listed in Table 1. We measured the distribution of the birds’ foraging aggregations at the biggest hotspot in front of Storbreen (more than 60% of water contact points in Brepollen) using the overlap of minimum convex polygons (MCP)^28^ of foraging ranges. The mean foraging range was 0.012 ± SE 0.003 km^2^ in area. The MCP overlap was more than 90% near the subglacial discharge of Storbreen and formed a hotspot about 40 m in radius, while an overlap of over 50% covered an area with a radius of about 100–120 m (Fig. 6c). The area inside the 90% contour is 0.007 km^2^, while that inside the 50% contour is 0.034 km^2^. Both areas lie completely within the discharge plume. Figure 6c also shows the concentration of water contact points in this area as the distribution of the number of points in a 5 × 5 m cell grid. We used the MCP method to compare the number of water contacts (which assume foraging) in Brepollen with other foraging areas. In Brepollen the MCP was delineated around all points (n = 1068). We used the Kernel Density method for all points outside Brepollen to choose the same number of points from the areas with the highest densities. Then the MCP was delineated around these 1068 points with the highest densities outside Brepollen and these continuous surface areas were compared: 54.7 km^2^ in Brepollen compared with 4 800 km^2^ outside.

## Discussion

Recent interest in bays with tidewater glacier termini has been generated mainly by the widely observed rapid retreat of glaciers and calving flux resulting from global warming: rising sea temperature accelerate ice melting. In this paper we describe the physical and biological features of the water in the vicinity of subglacial discharges and the mechanism by which unique sites functioning as foraging hotspots for seabirds are formed. They are very small in area and, as they attract thousands of birds feeding simultaneously, the foraging efficiency in them appears to be extremely high. That birds concentrate their foraging efforts in front of glaciers was known^13^, but the scale of the phenomenon was not. The spots we investigated were less than 54.7 km^2^ in area: this is equivalent to an area of 4800 km^2^ compared to the same amount of time the kittiwakes from one colony spend foraging on other feeding grounds during one breeding season. The same number of body contacts with water were recorded in both areas.

**Figure 7:**
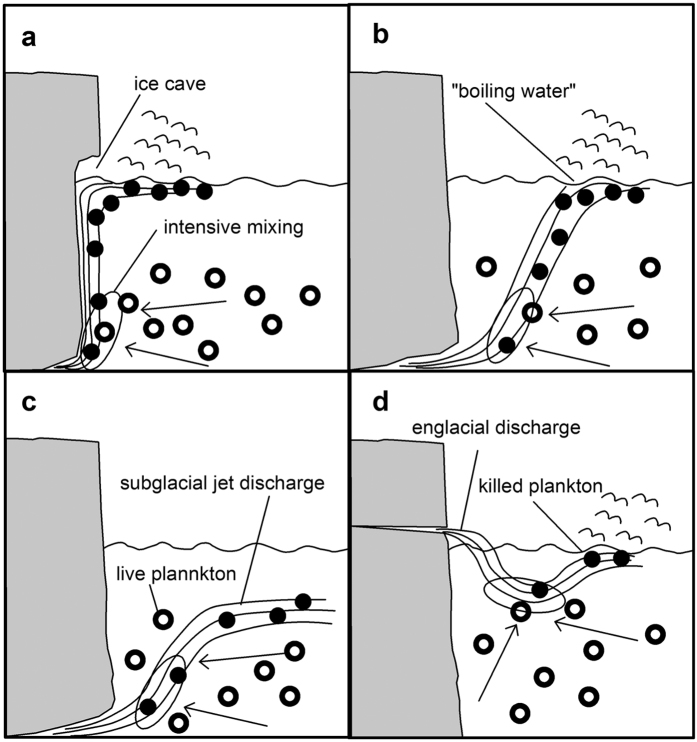
Subglacial discharges and foraging macroplankton hotspots. The subglacial discharge is due to the large entrainment velocity and creates an intensive mixing mechanism near the conduit, killing the macroplankton (**a**–**c**). The rising water - a buoyancy plume - delivers this plankton to the surface (**a**,**b**). When the water flows close to the ice face, it increases the melting rate and helps to create ice caves (**a**). The rising plume with plankton may also reach the surface at some distance from the ice front (**b**), or the plume does not reach the surface at all when it meets a density of water equal to that of the plume (**c**) Occasionally the englacial discharge may create a hotspot (**d**).

Tidewater glaciers can be classified as trunk or side-entry glaciers. It is worth mentioning that ever since glacier retreat has become widespread, many glaciers that in the past were classified as side-entry are now trunk glaciers. This has consequences for the local hydrology, as trunk glacier bays are a very low-energy environment^5^. The most important process, making these bays unique, is glacier meltwater discharge. This may be of three types – subglacial, englacial or supraglacial – to which we can add the subaerial rivers often present on both sides of glaciers. The meltwaters from all types are usually very turbid. These subglacial discharges occasionally create a mechanism by which zooplankton becomes concentrated (Fig. 7). The mass of the glacier exerts a pressure on the waters of a subglacial conduit that exceeds the hydrostatic pressure. Such pressures may give rise to water velocities much higher than those in open channels. The mechanism by which a forced plume entrains ambient fluid is described by the entrainment hypothesis^29^. According to this concept, which introduces the idea of entrainment velocity, the volume of entrained water is proportional to the mean central velocity and inversely proportional to the conduit radius. This creates a very efficient local mechanism of water mixing. One can estimate that in Raudvika, assuming a subglacial discharge of about 40 m^3^ s^−1^, the whole volume of water in the bay will be affected by this mechanism in one or two weeks. The consequence of such an event is that all the zooplankton are stunned or killed (the abrupt drop in salinity to <24 PSU has been found fatal for most of the local zooplankton^21^,^23^) and carried to one small area on the water surface. Since the entrainment velocity is much larger close to a conduit, it will be mainly zooplankton from layers of a similar depth that are transported to the surface. We observed, however, that not all subglacial discharges created foraging hotspots. In Burgerbukta, for example, we found subglacial discharge plumes but no foraging hotspots. We regularly observed aggregations of Kittiwakes during our bird surveys in July 2015, but the number of water contacts did not reach the level assumed to indicate a hotspot. This could be due to the different efficiency of a subglacial discharge in providing readily available zooplankton at the surface: this can be explained by the part played by entrainment velocity that depends on overburden pressure, which is a result of numerous factors of glacier origin. Our results show that the intensity of entrainment fluctuates in time and space. There were probably no hotspots in Brepollen in 2014 but they were certainly in existence in 2015. We consider that such variability can be explained by the changes in the presence and position of a subglacial discharge, entrainment velocity and the quantity of macroplankton in the water column.

The potential ecological importance of meltwater discharges raises questions about their distribution and variability. Fluvial discharges and entrainment govern water structure and temperature, salinity and SPM distribution in glacial bays. We derived a formula for converting satellite recorded reflectance to SPM for a wide range of turbidity values. We did this using Landsat 8 images, but our formula should also work well with Sentinel 2 images because the matching bands are similar. Though mainly of inorganic origin, the SPM in the glacier bays may differ in its mineralogical components, which influence the colour of the water. We tested our formula in Burgerbukta, where the colours of the turbid water are different from those in Raudvika, and obtained RMS = 11 mg l^−1^. So the formula may be useful and reliable for the high values of SPM common to glacier bays. Maps of surface SPM distribution show considerable variability of turbidity in front of the glacier. Turbid meltwater discharges reaching the surface form the main turbidity pattern. However, muddy waters are accompanied by patches of much clearer water. As the Landsat 8 and Sentinel 2 images have respective spatial resolutions of 30 and 10 m, all the surface meltwater discharges are identifiable. The complex hydrology of glacier bays as defined by the forcing factor of subglacial discharge may change in time and space, creating on occasion very attractive foraging grounds for birds. This is possible, however, only if a subglacial discharge reaches the surface, the entrainment velocity at the conduits is high, the depth at terminus is at least several tens of meters and there is sufficient macroplankton in the entrainment layer. Remote-sensing methods can be used to identify potential foraging hotspots at the water surface. The question whether discharge plumes flowing below the surface can give rise to foraging hotspots for fish and marine mammals, as noted in Vestre Burgerbukta, remains speculative.

We found that tidewater glacier bays were important foraging areas for surface feeding seabirds, kittiwakes in particular. Such sites, rich in easily available food and situated in the fjord close to colonies, are used as supplementary/contingency feeding grounds by seabirds that otherwise forage outside the fjord^30^,^31^. For kittiwakes these areas are of great significance, at least temporarily. Such an opportunity for emergency feeding close to the colony when weather conditions beyond the fjord are bad may increase the breeding success of birds^32^,^33^,^34^ and buffer the adverse consequences of climatic and oceanographic changes^35^,^36^,^37^.

Depending on the stage of retreat, glacier bays have a different importance for marine birds and mammals. The most attractive foraging grounds are formed in deep, tidewater glacier bays, with strong meltwater discharges, which draw zooplankton from a large area, become denser and rise to the surface. Foraging conditions for seabirds deteriorate when the glacier terminus reaches the coastline and the glacier bay becomes shallow. A rapid decline in sea ice cover in the Arctic may have serious consequences for pagophilic species, and changes in the cryosphere may have a drastic effect on Arctic biota, including seabirds and marine mammals^38^,^39^. Climate-induced sea-ice shrinking and glacier retreat, considered in the context of the sea-ice contact zone used by marine birds and mammals being reduced, may cause their numbers to decline. Tidewater glacier bays are thus the last refuges for pagophilic arctic animals^13^,^40^,^41^,^42^,^43^,^44^,^45^.

## Methods

We selected two bays – Burgerbukta in Hornsund (32 hydrological and 8 macroplankton stations) and Raudvika in Kongsfjord (52 hydrological stations) – for the hydrological and macroplankton studies. *In situ* measurements were made at the same time as the Landsat 8 satellite images were taken.

### GPS tagging and tracking study of kittiwakes

The study took place in the Gnallberget colony in Hornsund during the chick-rearing period in July 2015. The number of kittiwakes breeding in the colony is not known but is believed to range from 1 000 to 10 000 pairs (Norwegian Polar Institute, unpubl. data). To investigate the location of kittiwake foraging grounds, range of foraging flights and flight speed during the chick-rearing period, miniature global positioning system (GPS) loggers (“Sterna”, Ecotone, Sopot, Poland; printed circuit board size 35 × 16 × 10 mm) were used to record time, position and instantaneous speed. We used data from the GPS loggers deployed on 12 birds. The birds were trapped on nests using a long pole with a loop of fishing line at the start of the chick rearing period (10–15 July). The GPS loggers were attached to the bird’s central tail feathers using 2 mm wide strips of Tesa tape (code 4965 – Tesa Tape Inc., Charlotte, NC, USA). The birds were then released after no more than 10 min of handling. The foraging behaviour of kittiwakes observed in the tidewater glacier bays revealed them to be swarming over the subglacial discharge, with rapid simultaneous nose-diving and plunging into the surface water in pursuit of rising prey. Such behaviour usually resulted in the entire body getting wet, including the tail, to which the logger was attached. Every attempt by the bird to catch a prey item was registered by the logger’s conductivity sensor (wet-dry switch, 1 Hz sampling rate) as a record of contact with water. The logger’s weight (including attachment = 7.5–8.5 g) was equivalent to 1.8–2.0% of the body mass of individuals from Spitsbergen (mean body mass ± SD of the 21 adults from Hornsund caught in the same period as the GPS-logger equipped individuals: 408.0 ± 30.0 g). Sampling started after the first contact with salt water and the sampling interval was set at 15 min. The field-tested accuracy of the GPS receiver was ±10 m for 95% of positions. The GPS-loggers used a bidirectional radio link with the base stations installed in front of the colony, enabling remote data download. To save battery power, the base station automatically switched off the loggers while they were within the download range of the base station. The loggers started to collect GPS positions again when the birds flew beyond that range. All registered point data relating to bird contacts with water were first inspected in GIS software to identify potential hotspots in the glacier bays. We defined hotspots as areas with more than 50 points. As these occurred only in Brepollen, subsequent analyses were limited to this area. Subsets of points (as a vector point layer) were created for two glacier bays with the termini of Storbreen and Hornbreen. The number of points in each of the two main hotspots were determined for each logger and compared with the total number of logger points with water contact. Then, the ratio of hotspot use to all foraging sites could be calculated for each bird equipped with a logger, and also the mean value of this ratio, which is an estimate of hotspot use by a colony. All the results were entered in a Table 1. To avoid autocorrelation of data, the time series of recorded locations of each bird were divided into trips from and back to the colony. According to the mean flight speed, direction of movement and occurrence of water contacts, the activity during each trip was divided into three parts: the flight from the colony to the feeding grounds, foraging episodes and the return flight to the colony. This approach is justified by the birds’ activity during breeding. Each foraging episode was then treated as a separate sample. All the foraging episodes in Brepollen (78 episodes of 9 birds) were identified and saved as a set of points with unique ID number. For each foraging episode we delineated minimum convex polygons (MCP) to define the foraging range. The MCP is a commonly used method in telemetry analyses of animal movements^28^. Owing to the small number of points in an event and its compactness, we used the 100% of points convention. The MCP size and time of each foraging episode was also determined. To analyse the overlap of foraging ranges we converted MCPs to raster grids with a 10 m spatial resolution. The raster value assigned to a rasterized MCP was determined by the time of the foraging episode. Then all rasters were added and normalized to the 0–1 range. Two contours −0.5 and 0.9 (50% and 90% of the weighted overlap) – were drawn. To analyse the density and distribution of body-water contacts in a hotspot, we used quadrat analysis to measure the number of points in a cell grid^46^. The whole area was covered by a grid with a regular mesh of square polygons (fishnet) with a cell size of 10 m. The number of water contact points falling inside was assigned to each cell.

All experiments with live kittiwakes were carried out in accordance with the guidelines of the Governor of Svalbard and were granted under the provisions of the Regulations for the larger protected areas and bird reserves in Svalbard #11d. The experimental protocol was approved by the Norwegian Animal Research Authority (NARA) (ID 6467).

### Macroplankton measurements

Macroplankton was sampled with a rectangular neuston net (30 × 50 cm opening; 0.5 mm mesh). The net was used to collect samples from the surface to 30 cm depth and was hauled for 200 m at a speed of 2 knots from a zodiac rubber dinghy. Four samples were collected in the vicinity (about 200 m) of a glacier cliffs, accompanied by control samples taken 0.5 km to 1 km away from the cliffs (Fig. 5). Samples were collected from the cod end and preserved in 4% formaldehyde solution, to be analysed a few months later in the laboratory (Supplementary Table S1).

### Physical oceanographic measurements

All measurements were made from a zodiac rubber dinghy. The STD profiles were obtained using a SD204 self-contained instrument (SAIV A/S) that measures and records water salinity, temperature, pressure and turbidity. The turbidity was measured using a backscatter sensor in FTU (Formazin Turbidity Units). The sensor was calibrated to SPM using a conversion formula derived from the linear relation between turbidity and SPM measured at the same spot (with R2 = 0.82). The density was calculated using the standard salinity, temperature and pressure formula, modified to include the increase of density with high SPMs. Field measurements of SPM were obtained from discrete water sampling at the surface. The concentration of suspended particulate matter (SPM; mg l^−1^), defined as the dry mass of particles per unit volume of seawater, was determined using the standard gravimetric method. The concentration was estimated by vacuum-filtering measured volumes of water samples onto pre-combusted (450 °C, 24 h), pre-weighed MN GF-5 filters (0.4 μm pore size). The amount of filtered water differed, depending on the SPM concentration, and generally ranged between 150 and 2000 ml. This was enough for a distinct change in filter colour. After filtration of a sample, the filter was rinsed with 30 ml of deionised distilled water to remove salt. Large organisms visible to the naked eye were removed from the filters. These were placed in Petri dishes and stored in a refrigerator until analysis in the laboratory. Each filter was then air-dried at 60 °C for 24 h and weighed to determine the total dry mass of SPM. The concentration was determined by dividing the total dry mass of SPM by the amount of filtered water. Some samples were analysed in at least in two replicates, depending on the size of the sample, and the mean value calculated. The bathymetry of Raudvika was measured using Valeport echosounder profiling.

### Satellite measurements

Two cloudless Landsat 8 satellite images covering the study area were acquired for the days when field measurements were carried out. Both Landsat 8 scenes were obtained from USGS Glovis and processed in the same way. First, the DN was converted to TOA reflectance with a correction for the sun’s angle according to the USGS Landsat 8 product instructions. Then, an atmospheric correction using dark object subtraction (DOS1) was carried out, assuming 1% surface reflectance from the dark objects. We used standard methodology when seeking the best relation between satellite image information and SPM measured at the water surface by testing the relations between different variables (a simple function of satellite image bands) and independent variable (SPM) transformation using Ordinary Least Squares (OLS). The ratio of bands 4 and 2 (OLI4/OLI2) in all the models tested represented the best significance with excellent stability compared to the other variables and was used to derive the formula for converting the satellite image to an SPM concentration map.

## Additional Information

**How to cite this article**: Urbanski, J. A. *et al*. Subglacial discharges create fluctuating foraging *hotspots* for sea birds in tidewater glacier bays. *Sci. Rep.* **7**, 43999; doi: 10.1038/srep43999 (2017).

**Publisher's note:** Springer Nature remains neutral with regard to jurisdictional claims in published maps and institutional affiliations.

## Supplementary Material

Supplementary Table S1

Supplementary Video S2

## Figures and Tables

**Table 1:** GPS tracking study of kittwakes in Brepollen in 2015.

Logger ID	All contacts with water (cww)	cww in front of Storbreen	% of cww in front of Storbreen	cww in front of Hornbreen	% of cww in front of Horrnbreen	cww in front of Storbreen&Hornbreen	% in front of Storbreen&Hornbreen
1	221	0	0	0	0	0	0
2	109	9	8.2	3	2.8	12	11
3	268	7	2.6	0	0	7	2.6
4	271	103	38	90	33	193	71
5	121	38	31.4	28	23.1	66	54.5
6	166	94	56.7	16	9.6	110	66.3
7	109	0	0	0	0	0	0
8	289	0	0	0	0	0	0
9	420	161	38.3	53	12.6	214	50.9
10	366	103	28.1	0	0	103	28.1
11	173	24	13.9	0	0	24	13.9
12	439	339	77.2	0	0	339	77.2

Mean = 31.29.

std = 29.232.
