# Supplementary material for: Subglacial discharges create fluctuating foraging hotspots for sea birds in tidewater glacier bays
Source: Sci Rep. 2017 Mar 7;7:43999. doi: 10.1038/srep43999 (PMC5339806; doi:10.1038/srep43999)
Supplement: Supplementary Table S1 [file srep43999-s1.pdf]

Suplementary material for manuscript submitted to *Scientific Reports*:

Subglacial discharges create fluctuating foraging *hotspots* for sea birds in tidewater glacier bays

Jacek Andrzej Urbanski* , Lech Stempniewicz , Jan Marcin Węsławski , Katarzyna Dragańska-Deja , Agnieszka Wochna , Michał Goc , Lech Iliszko

Table S1. Mean macroplankton biomass registered in surface samples close to (G1-G4) and away from (G1 control- G4 control) the glacier front, 38 samples, July 2015, Hornsund.

TAXON	Size class	Ind. Mean Weight class	G1	G1 control	G2	G2 control	G3	G3 control	G4	G4 control
	mm	mg	biomass	biomass	biomass	biomass	biomass	biomass	biomass	biomass
Aglantha digitale	15	50	7.1	12.5	13.3	20	8.3	16.7	12.5	0.5
Clione limacina	15	80	11.4	0	208.3	0	0,0	0	40	0.25
Copepoda	5	5	446.4	280	53.3	53	350.0	250.0	1875	125
Ctenophora	10	40	120.0	80	5.8	24	13.3	13.3	20	0.25
Eupagurus zoea	5	5	3.6	0	0	0	45.8	8.3	55	0
Gammarus sp.	10	12	0	0	1.7	0	0	0	3	0
Helicina limacina	10	10	0	0	6.7	0	1.7	6.7	5	0.25
Hippolitidae larvae	10	20	0	0	0	0	0	6.7	5	0.75
Hyas zoea	5	10	104.3	12.5	70.0	0	0	3.3	5	0
Hydromedusae	10	30	55.7	112.5	6.7	0	0	50.0	195	2.5
Hyperia sp	5	10	8.6	5	0	0	1.7	0	2,5	0.25
Mysis oculata	15	55	0	0	1.7	0	0	0	1155	0
Onisimus littoralis	5	10	0	0	0	0	0	0	20	0
Sabinea spetemcarinata larvae	10	10	0	0	17.5	0	0	3.3	0	0
Sagitta elegans	10	15	19.3	0	340.0	18	0	0	0	0
Sagitta elegans	20	40	171.4	110	13.5	8	1020.0	893.3	840	1.75
Themisto abyssorum	10	27	7.7	0	6.7	0	0	0	27	0.25
Themisto abyssorum	5	10	0	0	0	0	0	0	0	0
Themisto libellula	10	30	4.3	0	0	0	0	0	0	0
Themisto libellula	15	50	0	0	15.0	0	8.3	0	12.5	0
Thysanoessa inermis	10	15	0	0	113.3	0	150.0	30.0	22.5	0
Thysanoessa inermis	20	85	12.1	0	10.0	0	255.0	28.3	0	0
Thysanoessa rashii	10	15	0.0	0	0	0	47.5	0	3.75	0
fish larvae	15	50	0.0	0	0	0	8.3	0	37.5	0
			972	613	884	123	1910	1310	4336	132

Video S2

Foraging hotspot of kittywakes in front of Storbreen, Hornsund (77.09691 N, 16.35786 E). Video recorded digitally by Michał Goc on 20 July 2016 using drone (DJI Phantom 2 Vision) with remote controlled camera. Note behavior of birds, brown water color and sea surface turbulence (boiling water).
